# Characterization of Cellulose Synthesis in Plant Cells

**DOI:** 10.1155/2016/8641373

**Published:** 2016-05-25

**Authors:** Samaneh Sadat Maleki, Kourosh Mohammadi, Kong-shu Ji

**Affiliations:** Co-Innovation Center for Sustainable Forestry in Southern China, Nanjing Forestry University, Nanjing 210037, China

## Abstract

Cellulose is the most significant structural component of plant cell wall. Cellulose, polysaccharide containing repeated unbranched *β* (1-4) D-glucose units, is synthesized at the plasma membrane by the cellulose synthase complex (CSC) from bacteria to plants. The CSC is involved in biosynthesis of cellulose microfibrils containing 18 cellulose synthase (CesA) proteins. Macrofibrils can be formed with side by side arrangement of microfibrils. In addition, beside CesA, various proteins like the KORRIGAN, sucrose synthase, cytoskeletal components, and COBRA-like proteins have been involved in cellulose biosynthesis. Understanding the mechanisms of cellulose biosynthesis is of great importance not only for improving wood production in economically important forest trees to mankind but also for plant development. This review article covers the current knowledge about the cellulose biosynthesis-related gene family.

## 1. Introduction

Plant cell wall is required not only for the structure to determine the actual shape of cells and functional properties to control inner and outer cellular communication but also for overall growth and tree expansion. Cellulose is the most significant component of plant cell walls. Due to the enormous economic beneficial aspect of tree cellulose for paper, lumber, pulp, and industrial products, understanding the mechanism of cellulose biosynthesis is valuable research objective.

## 2. Molecular Features of Cellulose

Cellulose is a polymer of glucose (C6H12O6), rotation 180° of one glucose molecule in relation to the next glucose to form *β* (1-4)-linked residues, called cellobiose (C12H22O11). Cellulose chain is made up of a repeating unit of cellobiose [1]. Cellulose is synthesized by cellulose synthase enzymes (CesAs) [2]. Cell walls consist of three types of layers. The middle lamella is formed during cell division as a first layer. The cell wall is microfibril-based; the primary cell wall (Pcw) is formed after the middle lamella but the secondary cell wall (Scw) is formed after cell enlargement completion. The secondary wall is often layered to S1, S2, and S3 (outer, middle, and inner layers, resp.) which vary in microfibrils orientation. S2 is the thickest layer with steep helices of microfibrils, while S1 and S3 are arranged in flat fibrillar slope [3]. All the cell wall layers consist of microfibrillar and matrix phases. The microfibrils have a crystalline core and less crystalline outer side but the matrix is noncrystalline phase that contains pectins and hemicelluloses, lignin, and other polymers [4]. The six crystalline polymorphs of cellulose, namely, I, II, III_I_, III_II_, IV_I_, and IV_II_, have been known. Cellulose I and cellulose II are the most common forms of cellulose, whereas others are not yet known to exist freely in nature. Cellulose I or native cellulose has two suballomorphs I*α* and I*β*. Both of them are found in higher plants [1]. The intramolecular hydrogen bonds are responsible for rigidity and stability of cellulose. Cellulose microfibrils I that have strong intra- and interchain hydrogen bonds which make the cellulose structures I and II run parallel and antiparallel to the long axis, respectively [5]. The degree of polymerization (DP) is different in Pcw and Scw which represent the monomer units in each cellulose chain which shows low DP, 2000–6000 for the primary cell wall, and high DP, 14000 in secondary cell wall [6].

## 3. History and Chromosomal Location of* CesA*

The CesA was initially identified in the Gram-negative bacterium* Acetobacter xylinus* [7]. In 1996, the plant CesA proteins were first recognized in ESTs isolated from cotton based on sequence homogeny to bacterial CesA [8]. CesA proteins have been localized to the plasma membrane. The* Arabidopsis* genome hosts a large family of cellulose biosynthesis genes, ten* CesA* genes (*CesA*1–10) and 29* Csl* encoding cellulose synthase-like genes [9]. Expression analysis of* Arabidopsis* revealed that CesA genes are expressed in most plant organs with some differences at the tissue level [10].

## 4. Structural Features of* CesA*

**Figure 1:**
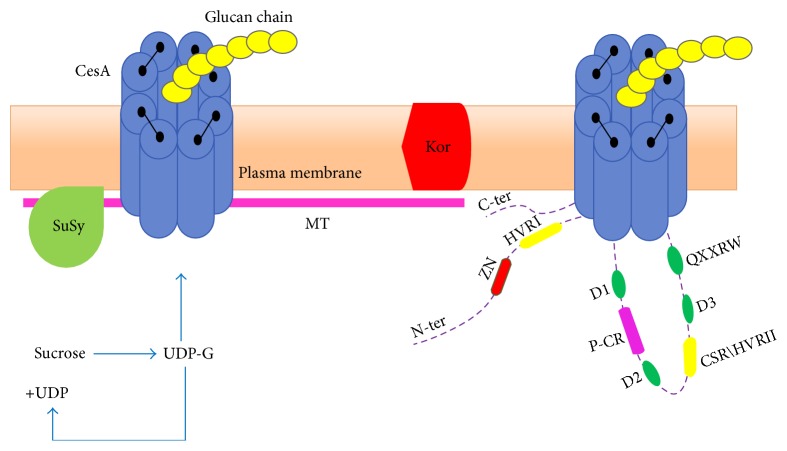
Model for structure of CesA proteins (right) shows a conserved zinc finger domain (ZN) and a hyper variable region (HVRΙ) near N-ter of TM1-2 and short C ter of TM3–8 and central hypervariable region (HVRΙΙ), plant-specific conserved region (P-CR), and class specific region (CSR); position of the processive glycosyltransferase motif D,D,D,QXXRW. Plant's cellulose biosynthesis (left). The plasma membrane-associated sucrose synthase (SuSy) channels uridine diphosphate-glucose (UDP-G) substrate to form rosette and glucan chain formation, the UDP formed can be recycled back to SuSy, and Korrigan cellulase (Kor) has been involved in monitoring of cellulose synthesis. Microtubules (MT) play role to regulate CesA proteins trafficking [2, 54, 72].

Different range of size* CesA* genes and amino acids in* Arabidopsis* is from 3.5 to 5.5 kb and 985 to 1088, respectively [11]. CesA is relevant to membrane-bound glycosyltransferase family 2 (GT-2) enzyme [12]. Glycosyltransferase is situated on the cytoplasmic domain between two sets of transmembrane domain. Eight TMDs are identified in plant CesA protein, TM1-2 towards N-terminus and TM3–8 towards C-terminus. These TMDs are suggested to form a pore across the inner membrane to embed cellulose chain secretion through the cell wall [13, 14]. In the conserved region, D,D,D,QXXRW, the first two ASP(D) residues help coordinate the uridine diphosphate, while the third D is the catalytic base and the QXXRW motif (Q is glutamine, R is arginine, W is tryptophan, and X is any amino acid) [10] (Figure 1) helps form a binding site for the terminal disaccharide of the growing glucan chain [15]. There is specific extra protein structure only in plants that consist of two (A, B) regions in GT domain: (A) highly conserved domains which have been named plant-specific conserved region (P-CR) and (B) specific conserved region and hypervariable region (HVR) which have been called class specific region (CSR) together [16] which sit in the mid part of GT (Figure 1). Sethaphong et al. [12] noted the CesA hexamer and tetramers assemblies are possible role of plant-specific subdomains and the CSR has highlighted role for dimers and trimmers assembly.

A comparison between bacterial and plant* CesAs* shows that a short N-terminal region contains 12 amino acids and 208 amino acids long in C-terminus of BcsA, while plant* CesAs* have short 17–21 amino acids long in C-terminus and 160–260 amino acids long in N-terminal region with zinc finger; 50 amino acids long is settled in this region [16]. Kurek et al. in 2002 proposed that the N-terminal zinc-binding domain partakes in protein interaction for rosette assembly in cotton; two* GhCesA1* and* GhCesA2* zinc finger domains were shown to interact with each other to regulate* CesA* assembly through dimerization via intermolecular disulfide bonds under oxidative conditions [14].

## 5. The CSC Models

As early as 1972, cellulose synthase complexes were visualized by electron microscopy [17]. Since CSCs were attached to the end of microfibrils and were observed in three rows ordered particles in alga* Oocystis apiculata*, so Brown Jr. and Montezinos [18] called them the linear terminal complex (TC) for the first time. Four years later, not only the connection between the rosette and the TC to synthesize microfibrils in higher plants was described by freeze fracture for first time, but also a different form of cellulose synthesizing sites was found as hexameric rosette TCs. Measurements suggest that rosette is 24 ± 2.5 in diameter, containing six particles with each of them having six cellulose synthase polypeptides to polymerize six glucan chains [19]. Cellulose synthase utilizes sitosterol glucoside which is synthesized by UDP-glucose as substrate to synthesis microfibril [20]. Ding and Himmel [21] proposed the cellulose microfibril model containing 36 glucose chains which is composed of both crystalline and noncrystalline chains by using atomic force microscopy (AFM) of direct visualization of the maize stem. In studies of the primary cell wall, except 36-chain model, two alternative models which apply to structures of CSCs containing the 24-chain and 18-chain models have been described. The 24-chain (eight three-chain sheets) models, three* CesA* polypeptides, make a particle and eight particles make a rosette formation with conformational disorder surfaces rather than packing disorders. The 18-chain twinned microfibrils (six three-chain sheets) models described the rosette with six particles of three cellulose synthase polypeptides. As the cross-sectional area of 36 chains microfibrils were evidently larger than the primary wall microfibril, so this model was a poor fit to experimental data. But 18-chain microfibrils model showed good fits to experimental data [22–24]. The rosette TCs are believed to be assembled by multiple* CesA* in the Golgi and then transported to plasma membrane in active form to cellulose synthesis by cytoplasmic vesicles [2, 25] which are termed SmaCCs (Small CesA Compartments) or MASCs (Microtubule Associated Cellulose Synthase Compartments), but then these small compartments have an operation in recycling CesA proteins from the plasma membrane [16, 26]. Multiple glucan chains can be synthesized by multiple cellulose synthase genes in each TC [27]. The rosette takes part in both glucan chain polymerization and crystallization [13].

## 6. Bacterial and Arabidopsis Genes That Encode Proteins of the Cellulose Synthase Complex

Bacterial cellulose synthase (Bcs) complex operon encodes four genes, BcsA, B, C, and Z. The Bcs A, B, and C activity is required for the synthesis and translocation of the polysaccharide; BcsZ encodes a cellulase to cellulose production [28–30]. The recent study on cellulose synthase operon genes (bcsABZC) of* Cronobacter* species confirmed the particular role of bcsA and bcsB mutants in cellulose production and showed involvement in biofilm formation and cell aggregation [31]. The cellulose synthesis in plants takes place in the context of rosettes more than TCs row [2] containing multiple steps, *β*-1,4-glucan chain initiation, elongation, and termination. Omadjela et al. [30] described that there is no requirement for a primer for chain initiation and there is no need to add other energy sources to the assembly of individual chains into a higher-order structure for cellulose synthesis in plants because the polymerization of UDP-glucose (DP range: 200–300) provides energy for growing cellulose chain through the membrane pore. Expression of different genes in* Arabidopsis* demonstrated that* AtCesA4*,* AtCesA7*, and* AtCesA8* are required to make the secondary cell wall, while* AtCesA1, AtCesA3,* and* AtCesA6* take part in cellulose biosynthesis of primary cell wall [32, 33].* AtCesA2, AtCesA5,* and* AtCesA9* appear to be partially redundant with* AtCesA6* [27]. No precise role has been assigned to* AtCesA10* [16]. First experimental evidence for the* CesA* function involvement in cellulose synthesis came from* Arabidopsis* mutant radial analyses swelling 1 (rsw1) which exhibited deficiency in cellulose content and number of rosette TCs at higher temperature in the primary cell wall due to changing valine amino acid to alanine in* AtCesA1* [34] and irregular xylem (irx) which represented defect in secondary cell wall formation in tissue xylem; in fact the* irx1 (CesA8), irx3 (CesA7), and irx5 (CesA4*) mutants exhibit collapsed or irregular xylem cells and reduced mechanical strength in fiber cells due to changing aspartic acid to asparagine, displacing tryptophan with a stop codon at position 859 and stop codon at position 263 replacing a glutamine, respectively [32, 35, 36] (see Table 1); observation of collapsed xylem cells resulting from mutation in* Exigua (exi*) genes, which were mapped to three cellulose synthase subunits* CesA4, CesA7*, and* CesA8*, leads to blocking water transport and reduced cell enlargement subsequently enhanced tolerance to osmotic stress which affects secondary cell wall deposition [37];* Mur10* mutants altered primary cell wall carbohydrate composition in response to secondary cell wall defects due to a mutation in* CesA7* locus [38]. Mutations in* CesA8* (lew2) enhance drought stress and accumulate ABA in secondary cell wall [39]. Stork et al. [40] reported the definite role for* CesA9* in* Arabidopsis* seed coats;* CesA9* mutant seeds contained 25% cellulose reduction and no changes in other tissues. Carroll et al. [41] in an analysis of transgenic lines in* Arabidopsis* demonstrated that* CesA7* and* CesA1* can rescue sort of deficiency in SCW biosynthesis in* cesa3* and in PCW biosynthesis in* cesa8ko* mutant, respectively.* IXR1* and* IXR2* mutant alleles are point mutations in the* CesA3* and* CesA6* genes that confer isoxaben resistance [42, 43];* CesA1aegeus* and* CesA3ixr1-2* mutants showed considerably reduced crystallinity and increased CesA velocity in the PM and resistance to quinoxyphen was conferred by* CesA1aegeus A903V* [44]; conversely, reduction in CSC velocity was observed in anisotropy1 D604N missense mutation in* CesA1* [45];* prc1-1* null* CesA6* mutant caused cellulose deficiency resulting in reduced cell elongation which was examined in* Arabidopsis* [33]. Surprisingly, both cellulose content reduction and constitutive stress response are due to accumulation of JA and ethylene in the* cev1* mutant in* CesA3* [46]. Lignification in nonlignified cells in eli1-1 and eli1-2* (CesA3*) inhibits cellulose synthesis that invokes overproduction of jasmonate and ethylene [47]. Reduction in primary and secondary cell wall thickness and cellulose content are affected by a missense mutation which occurred in fragile fiber 5 (*fra5*), a dominant mutant of* AtCesA7*, while neither cell wall thickness nor cellulose content is affected in* fra6* mutant form of* AtCesA8* [48].

## 7. Non-*CesA* Genes Involved in Cellulose Biosynthesis

Interestingly, apart from CesA proteins, the* KORRIGAN*, sucrose synthase (*SuSy*), microtubules and actin cytoskeletons, and* COBRA-like* proteins are involved in cellulose biosynthesis indirectly (Figure 1). It is thought that the membrane-bound endo-1,4-*β*-D-glucanase (KOR) has editing and monitoring role in conversion of glucan chain to release of newly synthesized cellulose microfibrils and eliminating defective glucan chains from the microfibril assembly [49, 50]. In theory, mutation in* KOR* may alter crystallization of the cellulose microfibrils [51]. Liebminger et al. [52] mentioned that activation of* A. thaliana KOR1* depends on utilization of eight N-glycosylation sites in the extracellular domain.* PtrKOR1* and* GhKOR1* get involved in secondary cell wall cellulose formation in* Populus tremuloides*, endosperm cellularization, and embryo development in cotton (*Gossypium hirsutum*) through RNAi suppression [53].

The strategic role of plasma membrane-associated sucrose synthase (*P-SUSY*) of developing cotton fibers (*Gossypium hirsutum*) in channeling UDP-glucose to cellulose synthase from sucrose was first illustrated in 1995 [54]; the UDP formed from UDP-G can be recycled back to SUSY (Figure 1). Mutation in SUS (1–4) in* Arabidopsis* shows less* SuSy* activity in all cells not in phloem and amazingly no cellulose deficiency was found, while the* sus5/sus6* mutants showed callose reduction in plates screening, but the plant growth was severely affected by mutant* invertase (INV),* recommending that catalysis of sucrose may need cytosolic invertase rather than SuSy [55]. But after two years Baroja-Fernández et al. [56] evidenced the possible role of sucrose synthase activity in cellulose biosynthesis under optimum pH 7.0 conditions.

The relation between the cytoskeleton (cortical microtubules and actin) and CSC localization and movement has been the topic in the majority of studies. MTs are another key player in plant cell morphogenesis. Gardiner et al. [57] showed the colocalization of all three AtCesA4 (IRX5), AtCesA7 (IRX3), and AtCesA8 (IRX1) proteins with cortical microtubule bands in older developing xylem vessel with GFP. But actin microfilaments localize with the CesA proteins to regions of cell wall thickening. Intracellular trafficking and plasma membrane localization of the CSC during secondary cell wall formation were supported by live-cell imaging of fluorescently labeled proteins fusion [58–63]. The alignment hypothesis describes that microtubules can control the alignment of cellulose microfibril deposition in the plasma membrane [59, 61]; however, the examination results of polymerization and depolymerization of MTs with short treatment by taxol and oryzalin, respectively, do not agree with the alignment hypothesis because no changes were observed in cellulose microfibril orientation [60]. Li et al. [61] investigated the role of CesA interactive protein 1 (CSI1) as a linker protein in association between CesA complexes and cortical microtubules* in vivo*. Based on result of Zhong et al. [62] the* FRA1, kinesin-like* microtubule binding protein, mutant made alteration deposition of cellulose microfibrils in fiber cell walls in* Arabidopsis*. Intracellular trafficking of CSC by actin filaments has been suggested by Wightman and Turner [63] for controlling delivery of CSC to the PM to maintain proper patterned deposition of the Scw. Newly, dynamic coordination between AF and MT was investigated using dual labeled probe in interphase plant cell [64].

A role of* COBRA *(*cob*) mutant in regulating the orientation of cell expansion was associated with a screen for* Arabidopsis* with defect expanded roots for the first time in 1993 [65]. Short and swollen roots resulted from mutations in the* COB*. It encodes a putative GPI-anchored protein that is necessary for oriented cell expansion in* Arabidopsis*.* COBRA* causes the cell elongation and reduction in crystalline cellulose content. Also it has been identified in association with deposition of cellulose microfibrils in the root tissue [66, 67].* COBRA-like* gene family members* COBL2, COBL6, COBL9, COBL10,* and* COBL11 *are required for oriented crystalline cellulose deposition during seed development, root hairs, and pollen tube elongation in PCW, respectively, while* COBL4* was identified during the formation of the vascular system in xylem cells [68, 69]. As* irx6* is a member of the COBRA gene family (*COBL4*), it also regulates continuous growth cell and manifests decreased crystalline cellulose content in root cell walls [70, 71].

## 8. Conclusion

Despite the numerous studies and progress towards understanding the mechanism of cellulose biosynthesis in higher plants, numbers of questions are still pending. How many proteins are necessary for cellulose synthesis process? How can every plant regulate the synthesis of cellulose? How is the CesA position in cellulose synthesis complex? How does a plant cell control the crystallization of microfibrils? Investigation of cellulose structure and key genes engaged in cellulose synthesis can be important because of widespread utilization of cellulose product in daily life.

## Figures and Tables

**Table 1:** *Arabidopsis* CesA mutants and their phenotypes.

Gene name	Mutant alleles	Phenotype	References
*CesA1*	*rsw1-1 A549V* *aegeus A903V* *any1 D604N*	Deficiency in cellulose content and number of TCs Quinoxyphen resistant, modified cellulose crystallinity Reduction in cellulose crystallinity and CSC velocity	[34] [44] [45]

*CesA3*	*ixr1-1G998D* *ixr1-2 T942*	Isoxaben resistance	[42]
	*cev1 G617E*	Constitutive expression of JA and ethylene	[46]
	*eli1-1 S301F* *eli1-2 A522V*	Reduced cellulose synthesis, activating lignification and defense responses	[47]

*CesA4*	*irx5-1* *irx5-2 W995stop* *irx5- 3 Q263stop*	Irregular xylem, defective cellulose biosynthesis, dwarf plants	[32]
	*exi2 Y939stop*	Vascular defect, cell expansion defect, collapsed xylem, small rosette leaves, reducing the cell expansion	[37]

*CesA6 *	*prc-19Y 275 STOP* *prc1-4/5W 777 STOP* *prc1-9K 7222 STOP* *prc1-1/3Q 720 STOP*	Stunted hypocotyl and roots; incomplete cell wall	[33]
	*ixr2-1 R1064W*	Resistance to isoxaben and semidominant allele	[43]

*CesA7*	*irx3 W859stop* *fra5 P557T* *mur10-1 W444stop* *mur10-2 H734Y*	Irregular xylem and defective cellulose biosynthesis Reduced fiber cell wall thickness and cellulose content No deposition of secondary wall	[35] [48] [38]
	*exi5 W954stop*	Vascular defect, collapsed xylem, small rosette leaves, reducing the cell expansion	[37]

*CesA8*	*irx1-1 D683N* *irx1-2 S679L*	Irregular collapsed xylem and defective cellulose biosynthesis	[36]
	*fra6 R362K*	Recessive allele	[48]
	*lew2-1 W217stop* *lew2-2 L792F*	Leaf wilting, disruption of cellulose synthesis in SCW, increased tolerance to drought and osmotic stress	[39]
	*exi1-1 splicing variant* *exi1-2 G508E*	Vascular defect, cell expansion defect, collapsed xylem defect, small rosette leaves	[37]

## Abbreviations

CesA: Cellulose synthase catalytic subunit
Csl: Cellulose synthase-like
Pcw: Primary cell wall
Scw: Secondary cell wall
GT: Glycosyltransferase
TMD: Transmembrane domain
HVR: Hypervariable region
TC: Terminal complex
CMF: Cellulose microfibril
Kor: Korrigan cellulase
MT: Microtubule
SuSy: Sucrose synthase
UDP-G: Uridine diphosphate-glucose
GFP: Green fluorescent protein.
